# Patient and allograft outcomes after kidney transplant for the Indigenous patients in the United States

**DOI:** 10.1371/journal.pone.0244492

**Published:** 2021-02-03

**Authors:** Regan Seipp, Nan Zhang, Sumi Sukumaran Nair, Hasan Khamash, Amit Sharma, Scott Leischow, Raymond Heilman, Mira T. Keddis

**Affiliations:** 1 Division of Nephrology and Hypertension, Department of Medicine, Mayo Clinic, Phoenix, Arizona, United States of America; 2 Department of Health Science Research, Section of Biostatistics, Mayo Clinic, Phoenix, Arizona, United States of America; 3 Division of Dermatology, Department of Medicine, Mayo Clinic, Phoenix, Arizona, United States of America; 4 Office of Health Care Disparity, Mayo Clinic, Phoenix, Arizona, United States of America; Weill Cornell Medicine, UNITED STATES

## Abstract

**Background:**

The objective is to assess cardiovascular (CV), malignancy, infectious, graft outcomes and tacrolimus levels for the Indigenous patients compared to Whites after kidney transplant (KTx).

**Methods:**

165 Indigenous and 165 White patients matched for the KTx year at Mayo Clinic Arizona from 2007–2015 were studied over a median follow-up of 3 years. Propensity score was calculated to account for baseline differences.

**Results:**

Compared to Whites, Indigenous patients had the following characteristics: younger age, more obesity, diabetes, hypertension, and required dialysis prior to KTx (p<0.01). Indigenous patients had longer hospital stay for KTx, shorter follow-up and lived further from the transplant center (p<0.05). 210 (63.6%) received deceased donor KTx and more Whites received a living donor KTx compared to Indigenous patients (55.2% vs 17.6%, p<0.0001). Post-KTx, there was no difference in the CV event rates. The cumulative incidence of infectious complications was higher among the Indigenous patients (HR 1.81, p = 0.0005, 48.5% vs 38.2%, p = 0.013), with urinary causes as the most common. Malignancy rates were increased among Whites (13.3% vs 3.0%, p = 0.001) with skin cancer being the most common. There was a significant increase in the dose normalized tacrolimus level for the Indigenous patients compared to Whites at 1 months, 3 months, and 1 year post-KTx. After adjustment for the propensity score, there was no statistical difference in infectious or graft outcomes between the two groups but the mean number of emergency room visits and hospitalizations after KTx was significantly higher for Whites compared to Indigenous patients.

**Conclusions:**

Compared to Whites, Indigenous patients have similar CV events, graft outcomes and infectious complications after accounting for baseline differences.

## Introduction

The indigenous patients in the United States experience disproportionally higher rates of obesity, diabetes and chronic kidney disease compared to Whites with resultant increased prevalence of end stage kidney disease (ESKD) and ESKD mortality outcomes [1–8]. Compounding these disparities, the Indigenous patients have lower access to kidney transplant (KTx) evaluation, are less likely to be approved for and be actively listed for KTx and have the lowest rates of KTx of any minoritized group [7,9–11]. For Indigenous patients who do undergo KTx, current evidence examining post-transplant patient and graft outcomes are limited.

Graft and patient survival were reported in cohorts ranging from 16–61 patients before the year 2000 [12,13] with one study showing inferior graft survival among the Indigenous patients and the larger study showing similar graft and patient survival despite greater histocompatibility mismatch (HLA). With the advent of new immunosuppression regimens and decline in the rates of allograft rejection over the years, understanding cardiovascular (CV), infectious and malignancy related morbidity for the Indigenous patients is necessary. Total cumulative immunosuppressive dose is well correlated with the development of malignancy and the development of opportunistic infections [14]. In the Indigenous patients, the metabolism of tacrolimus is decreased due to increased prevalence of CYP3A5 loss of function alleles. CYP3A5 is required for the metabolism of tacrolimus. Loss of function CYP3A5 leads to lower doses of tacrolimus in the Indigenous patients compared to Whites to achieve similar target levels [15,16]. Lower cumulative immunosuppression dosing may lower infection rates and lower malignancy events. Whether this reduced tacrolimus dosing affects post-transplant infection and malignancy rates in Indigenous patients has not been previously studied. Mayo Clinic Arizona transplant center is the largest transplant center with respect to transplant volume in ESKD Network 15 (one of 18 government funded networks for oversight of ESKD care) where the Indigenous patients represent 9.5% of prevalent dialysis patients [17], the highest in the United States and ranks in the top 10 centers in KTx volumes [18]. We have previously reported on the KTx evaluation process and outcomes for the Indigenous patients in the United States compared to Whites [10]. This is a follow-up study investigating post-transplant outcomes. The purpose of this study is to 1) evaluate the rates of CV, infectious, and malignancy related events, 2) graft and patient outcomes, 3) emergency room (ER) visits and hospitalizations, and 4) tacrolimus dosing for the Indigenous patients compared to Whites after KTx at Mayo Clinic Arizona.

## Methods

### Study population

The Mayo Clinic Institutional Review Board approved this study. The study was deemed to be low risk and patient consent was waived. An equal number of indigenous and whites who received a KTx from 2007 to 2015 at Mayo Clinic Arizona were included. Patients of Indigenous race were identified using the medical record and were restricted to non-Hispanic or Latino. The comparison group was comprised of an equal number of White non-Hispanic or Latino patients matched for the year of transplant. Matching for the year of transplant was necessary to account for changes in the transplant practice during that time period. Because the majority of patients evaluated for KTx at our center are white Americans, we used a computer generated random sampling process to populate a comparison group of white non-Hispanic or Latino American patients from the entire cohort transplanted from 2007–2015 and matched for the year of transplant. The number of randomly selected white Americans matched the number of the Indigenous patients per year of KTx. The study cohort included a total of 330 patients. We assessed 165 Indigenous American and 165 non-Hispanic white patients matched for the year of transplant between the years of 2007–2015. The number of patients included in this study was limited by the number of Indigenous patients who received a KTx at our institution during that time period. All patients received standard immunosuppression including tacrolimus (or cyclosporine in 5 patients), mycophenolate mofetil (or azathioprine in 6 patients) with or without chronic corticosteroid therapy.

### Patient variables

The following demographic and co-morbid conditions present at the time of transplant were analyzed: age, gender, diabetes, hypertension, dialysis requirement, body mass index (BMI), and length of hospital stay, prior kidney transplants, transplant donor type and race, number of human leukocyte antigen (HLA) mismatches and CV diseases. Cardiovascular disease was defined as any of the following: history of heart failure, coronary artery disease, cerebrovascular disease, and vascular disease. Coronary artery disease was defined as history of myocardial infarction, angioplasty, and/or coronary artery bypass surgery. Vascular disease was defined as requiring amputation or revascularization. Cerebrovascular disease was defined as either ischemic or hemorrhagic stroke. Medication use including statin therapy, aspirin and beta blockers was recorded.

Tacrolimus level at 1 months, 3 months and 1 year post-transplant and corresponding total prescribed doses were recorded and dose-normalized ratio was obtained. Dose-normalized trough level was calculated by the ratio of trough level (ng/mL) to cumulative daily dose (mg). For each patient, tacrolimus data was aggregated at each time point of interest.

### Outcome variables

We evaluated several post-kidney transplant outcomes that occurred at any time after KTx until last follow-up or for the duration of the study (November 2017).The following event rates were assessed: diabetes after transplant, CV events, infectious complications, malignancy, and graft outcomes. Infections were classified per organ system: gastrointestinal, genitourinary, pulmonary, or others. Infection outcome was defined as a composite of any of the infections per organ system. Infectious severity was defined as 1) localized, 2) with bacteremia or sepsis, or 3) shock state. Rates of polyomavirus (BK) and cytomegalovirus (CMV) rates were also reviewed and analyzed separate from cumulative infection outcome. Types of malignancy were identified based on primary site. Graft outcomes of interest included: rates of delayed graft function (defined as requiring dialysis within seven days of transplant), rates of acute rejection (defined based on allograft biopsy) and graft function based on serum creatinine measurements and estimated glomerular filtration rate (eGFR). Graft failure was defined as return to dialysis or requiring evaluation for another KTx. Lastly, we reviewed health care utilization defined by number of emergency room visits and hospitalizations at our institution during the first year post-transplant.

### Statistical analysis

Mean with standard deviation or median with range was used to report continuous variables and two sample t-test or Wilcoxon Rank Sum test was used to report comparisons of continuous variables. Categorical variables were reported as counts and proportions and compared using Chi-square or Fisher’s exact test. Cumulative incidences of outcomes of interest were estimated and compared between the two groups using Gray’s method in the presence of competing risk events (incidence of acute rejection with death as a competing risk). Propensity score was calculated based on clinically relevant variables based on outcomes of interest and statistically significant differences at baseline between the two groups with minimal number of missing variables and these included: age at KTx, gender, dialysis requiring, donor type, cancer history, hypertension, and, diabetes. Logistic regression modeling was used for binary variables and negative binomial regression for continuous variables. A proportional hazards model to estimate the hazard ratio for each potential predictor was utilized [19]. The modeling strategy included the following: each potential predictor was examined in a univariate manner and the factors that were clinically significant and those that were significant at 0.05 level were chosen into the final model. For analysis of tacrolimus trough level with or without dose-normalized ratio, mixed model using repeated measures was used to model the data and interaction with race and time post-transplant was evaluated. All statistical analyses were performed using SAS, version 9.3 (SAS Institute, Cary, NC).

## Results

### Baseline characteristics

Three hundred and thirty patients were studied, 165 Indigenous patients and 165 Whites matched for the KTx year over median follow-up period of 3 years (0.1–10.6). The mean age of the cohort studied was 55.3±13.1 years and 187 (56.7%) were male. Compared to Whites, Indigenous patients were younger (mean age 51.2±12.2 vs. 59.4±12.8 years, p<0.0001), had higher BMI (mean 30.4±5.9 vs 27.8±6.1 kg/m^2^, p = 0.0001), were more likely to have diabetes (n = 108 (65.5%) vs n = 36 (21.8%), p<0.0001) and hypertension (n = 153 (92.7%) vs n = 136 (82.4%), p = 0.005), require dialysis (n = 156 (94.5%) vs n = 101 (61.2%), p<0.0001) and longer duration of dialysis (median 49.6 (1.5, 263 months) vs 23 (0.37, 82.2 months), p<0.001). History of cancer was more prevalent among Whites than Indigenous patients (n = 46 (27.9%) vs n = 8 (4.8%), p<0.0001). There was no significant difference in the prevalence of CV events prior to transplant as shown in Table 1. Whites were more likely to have had a prior kidney transplant compared to Indigenous patients (n = 23 (13.9%) vs n = 10 (6.1%), p = 0.017). Indigenous patients had significantly shorter follow-up than Whites (median 2.0 (0.1–10.6) vs 3.0 (0.3–10.1) years, p<0.002) and lived further from the transplant center (median 125 miles (7.5, 341.0) vs 20 (0.0, 1953.7), p< 0.001). Two hundred and ten (63.6%) received a deceased donor KTx. Whites were more likely to receive a living donor KTx compared to Indigenous patients (n = 91 (55.2%) vs n = 29 (17.6%), p<0.001). The median hospital stay for the entire group was 3 days with the Indigenous patients having longer stays than whites as shown in Table 1.

**Table 1:** Baseline characteristics.

	Indigenous (N = 165)	White (N = 165)	Total (N = 330)	P-value
Age, mean (sd)	51.2 (12.2)	59.4 (12.8)	55.3 (13.1)	< .001^1^
Gender (male), n (%)	95 (57.6%)	92 (55.8%)	187 (56.7%)	0.739^2^
Body Mass Index (BMI, kg/m2), mean (sd)	30.4 (5.9)	27.8 (6.1)	29.1 (6.2)	< .001^1^
Previous kidney transplants, n (%)	10 (6.1%)	23 (13.9%)	33 (10.0%)	0.017^2^
Length of hospital stay for kidney transplant (days), median (min, max)	4 (2.0, 9.0)	3 (2.0, 10.0)	3 (2.0, 10.0)	0.015^3^
Dialysis, n (%)	156 (94.5%)	101 (61.2%)	257 (77.9%)	< .001^2^
Dialysis duration (days), median (interquartile range)	1550 (886, 2205)	184 (0, 822)	860 (119, 1685)	<0.001^3^
Donor type (deceased), n (%)	136 (82.4%)	74 (44.8%)	210 (63.6%)	< .001^2^
Malignancy, n (%)	8 (4.8%)	46 (27.9%)	54 (16.4%)	< .001^2^
Hypertension, n (%)	153 (92.7%)	136 (82.4%)	289 (87.6%)	0.005^2^
Diabetes, n (%)	108 (65.5%)	36 (21.8%)	144 (43.6%)	< .001^2^
History of cardiovascular events, n (%)	44 (26.7%)	51 (30.9%)	95 (28.8%)	0.395^2^
End stage kidney disease cause, n (%)				<0.001^2^
Diabetes	102 (61.8%)	24 (14.5%)	126 (38.2%)	
Glomerulonephritis	25 (15.2%)	34 (20.6%)	59 (17.9%)	
Polycystic kidney disease	4 (2.4%)	28 (17.0%)	32 (9.7%)	
Hypertension	7 (4.2%)	13 (7.9%)	20 (6.1%)	
Re-transplant	10 (6.1%)	22 (13.3%)	32 (9.7%)	
Other	17 (10.3%)	44 (26.7%)	61 (18.5%)	
Donor specific antibody, n (%)	23 (13.9%)	15 (9.1%)	38 (11.5%)	0.168^2^
Steroid free immunosuppression, n (%)	115 (69.7%)	88 (53.3%)	203 (61.5%)	0.002^2^
Follow-up after transplant (years), median (min, max)	2 (0.1, 10.6)	4 (0.3, 10.1)	3 (0.1, 10.6)	0.002^3^
Distance from transplant center (miles), median (min, max) 4	125 (7.5, 341.0)	20 (0.0, 1953.7)	112 (0.0, 1953.7)	< .001^3^

^1^Equal variance two sample t-test

^2^Chi-square p-value

^3^Kruskal-Wallis p-value.

Three different types of induction immunotherapy were utilized during the study period. Whites received more basiliximab (35.2 vs 7.27%) and Indigenous patients received more alemuzumab (60.6 vs 43.0%) and thymoglobulin (32.1 vs 21.8%), p<0.0001. More Indigenous patients did not receive corticosteroid therapy as part of maintenance immunosuppression than Whites (69.7 vs 53.3%, p = 0.002). There was a greater number of HLA mismatches for the Indigenous patients compared to Whites (4.02 ± 1.55 vs 3.52 ±1.75, p = 0.007).

### Kidney transplant outcomes

We evaluated CV, infectious, and malignancy outcomes between the two groups. There was no difference in the CV event rates between the two groups post-transplant. It should be noted that the use of statin therapy was less frequent among Indigenous patients compared to White at last follow-up (n = 44 (26.7%) vs n = 60 (36.4%), p = 0.058). There was no difference in aspirin use or beta blocker use. The rates of malignancy were significantly higher among whites compared to indigenous patients (n = 22 (13.3%) vs n = 5 (3.0%), p = 0.001). Skin cancer was the most common cancer among whites (7 cases of squamous cell carcinoma and 1 case of melanoma (total of 8) versus 0 among the Indigenous patients). There were 5 Indigenous patients who developed malignancy post-KTx (1: central nervous system lymphoma, 1: multiple myeloma, 1: pancreatic, 2: renal cell carcinoma). There were 22 White patients who developed malignancy (1: esophagus, 1: stomach, 2: lung, 1: myelodysplastic syndrome, 1: neuroendocrine tumor, 1: oncocytoma, 1: pancreatic, 2: prostate, 1: renal cell carcinoma, 2: squamous cell carcinoma of head and neck, 1 stomach, and 1 urothelial).

Infections were more common among the Indigenous patients than Whites (n = 80 (48.5%) vs n = 63 (38.2%)) and genitourinary causes were more prevalent among the Indigenous patients (n = 58 (35.2%) vs n = 29 (17.6%), p = 0.0132).

**Fig 1:**
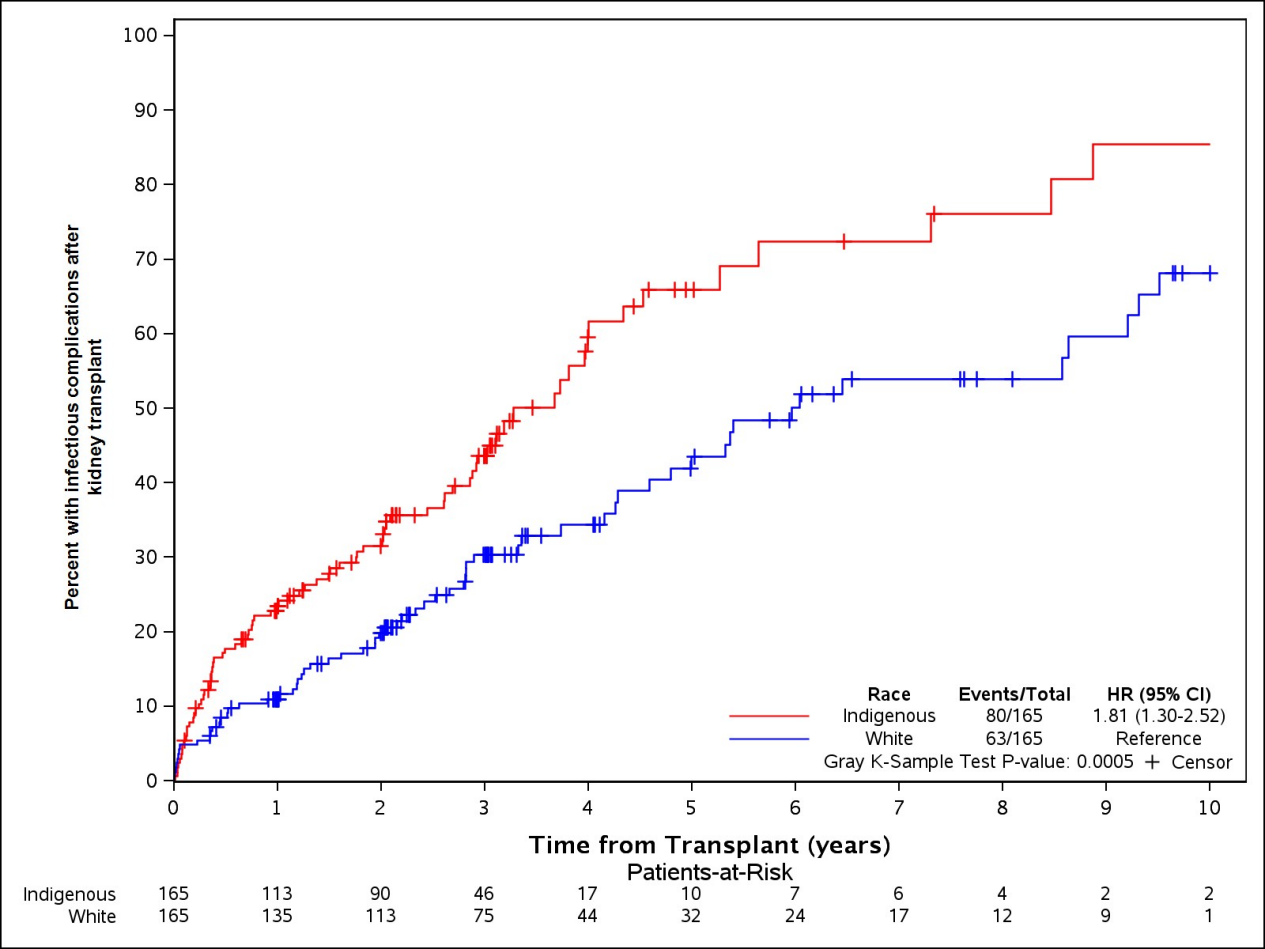
Cumulative incidence of post-kidney transplant infectious complications. The incidence of infectious complications was higher for Indigenous patients compared to Whites.

The cumulative incidence of post-transplant infections for the Indigenous patients was significantly higher compared to Whites (HR 1.81 (1.30–2.52), p = 0.0005) as shown in Fig 1. For the first year post-transplant, the incidence of infections was 23.5 (17.8–31.1%) vs 11.0 (7.1–17.0%) compared to whites. There was no difference in the rates or cumulative incidence of infectious severity, BK or CMV infection as shown in Table 2.

**Table 2:** Post-kidney transplant infectious complications.

	Indigenous (N = 165)	White (N = 165)	Total (N = 330)	P-value*
Cumulative infection rates				0.0590
	80 (48.5%)	63 (38.2%)	143 (43.3%)	
Infection type				0.0132
Gastrointestinal	2 (2.5%)	8 (12.7%)	10 (7.0%)	
Primary urinary	53 (66.3%)	27 (42.9%)	80 (55.9%)	
Urinary and lung	4 (5.0%)	2 (3.2%)	6 (4.2%)	
Urinary and other	1 (1.3%)	0 (0.0%)	1 (0.7%)	
Primary lung	10 (12.5%)	16 (25.4%)	26 (18.2%)	
Lung and other	0 (0.0%)	1 (1.6%)	1 (0.7%)	
Other	10 (12.5%)	9 (14.3%)	19 (13.3%)	
Infection Severity				0.8917
Missing	85	102	187	
Focal/localized	29 (36.3%)	27 (42.9%)	56 (39.2%)	
Bactremia	6 (7.5%)	4 (6.3%)	10 (7.0%)	
Bactremia & Sepsis	1 (1.3%)	0 (0.0%)	1 (0.7%)	
Sepsis	38 (47.5%)	29 (46.0%)	67 (46.9%)	
Shock	6 (7.5%)	3 (4.8%)	9 (6.3%)	
BK infection	39 (23.6%)	39 (23.6%)	78 (23.6%)	1.0000
CMV infection (including CMV viremia)	25 (15.2%)	21 (12.7%)	46 (13.9%)	0.6339

*: Wilcoxon rank sum test, Chi-square test or Fisher’s exact test was used when appropriate.

### Allograft and mortality outcomes

More Indigenous patients had delayed graft function after transplant (n = 71 (43%) vs n = 26 (15.8%), p<0.001). The rates of acute rejection were similar between the two groups but the first year post-transplant cumulative incidence was increased for Indigenous patients compared to Whites (15.4 (9.5–20.8%) vs 14.0 (8.6–19.2%), p = 0.046). In patient with deceased KTx, the first year cumulative incidence of acute rejection was no longer statistically significant but there remained a trend towards higher rates of acute rejection in the Indigenous patients (16.8 (11.4–24.7%) vs 11.1 (5.7–21.5%), p = 0.075). Forty nine (14.8%) patients had graft failure during the follow-up time. The cumulative incidence of graft failure between the two groups was similar. Thirty-five patients died. Overall survival was similar between the two groups (p = 0.096).

### Propensity analysis

After adjusting for propensity score (which accounted for differences in age, gender, dialysis at the time of KTx, donor type, pre-KTx malignancy, hypertension, and diabetes, and steroid free immunosuppression), there was no statistically significant difference in infectious (composite of all infections) or graft outcomes (acute rejection and graft failure free survival). The mean number of ED visits and hospitalizations after KTx remained significantly higher for Whites compared to Indigenous patients as shown in Table 3.

**Table 3:** Post-transplant outcomes on univariate analysis and after adjusting for propensity score.

Outcomes at last follow-up after transplant	Indigenous	White	P-value (univariate)^1^	P-value (adjusted)^2^
Statin, n (%)	44 (26.7%)	60 (36.4%)	0.059	0.577
Aspirin, n (%)	67 (40.6%)	53 (32.1%)	0.110	0.946
Beta blocker, n (%)	88 (53.3%)	77 (46.7%)	0.226	0.361
Diabetes, n (%)	110 (66.7%)	44 (26.7%)	< .001	0.848
Hypertension, n (%)	138 (83.6%)	124 (75.2%)	0.058	0.891
Cardiovascular events, n (%)	25 (15.2%)	30 (18.2%)	0.461	0.314
Infectious events, n (%)	80 (48.5%)	63 (38.2%)	0.060	0.903
Malignancy, n (%)	5 (3.0%)	22 (13.3%)	0.002	0.436
Emergency room visit rates after transplant, n (%)	99 (60.0%)	121 (73.3%)	0.011	0.018
Emergency room visits after transplant, mean (sd)	1.9 (3.5)	3.5 (5.1)	< .001	< .001
Re-hospitalizations rates after transplant, n (%)	99 (60.0%)	105 (63.6%)	0.497	0.153
Re-hospitalization after transplant, mean (sd)	2.0 (3.1)	3.2 (4.9)	0.008	0.002
Acute rejection, n (%)	35 (21.2%)	27 (16.4%)	0.261	0.193
Delayed graft function, n (%)	71 (43.0%)	26 (15.8%)	< .001	0.283
All-cause mortality, n (%)	15 (9.1%)	20 (12.1%)	0.373	0.878

^1^Univariate regression was used, only race was added to the model.

^2^Multiple regression model adjusted for race and propensity score (which accounted for differences in age, gender, dialysis at the time of transplant, donor type, baseline history of malignancy, hypertension, diabetes, and steroid free immunosuppression).

### Tacrolimus dose and trough concentrations between the two groups

**Fig 2:**
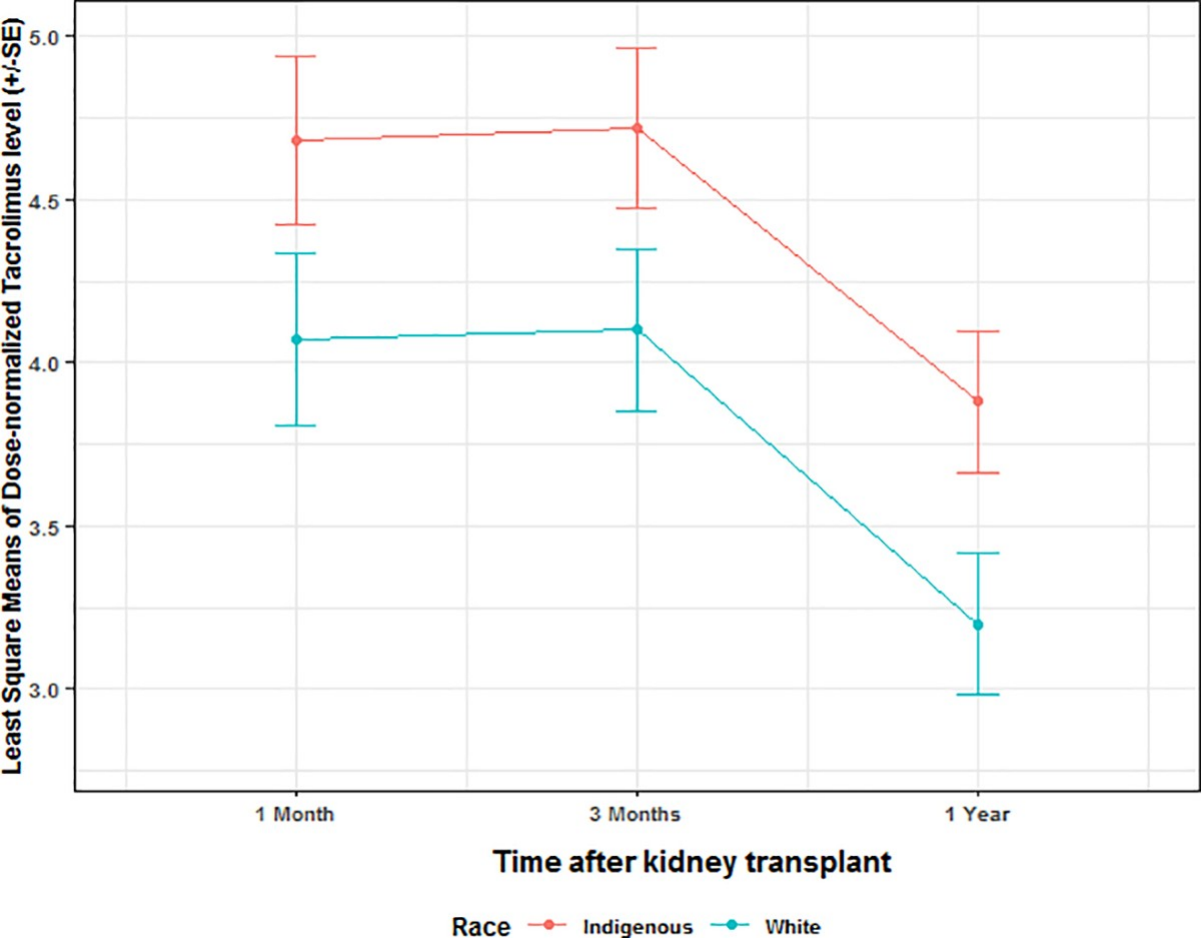
Dose normalized tacrolimus level between the two groups.

The median daily dose of tacrolimus was similar between the two groups (Indigenous: median 2 (0.0, 8.0) vs White: 3 (0.0, 12.0), p = 0.108). Tacrolimus trough level data by least square means was similar between the two groups at 1 months, 3 months, and 1 year post-KTx (p = 0.218). The trough level decreased significantly overtime but this trend was similar between the two groups (p = 0.389). Indigenous patients had significantly higher dose normalized tacrolimus level at 1 year compared to Whites (p = 0.028) as shown in Fig 2.

Dose normalized tacrolimus level was significantly higher in the Indigenous patients compared to Whites at 1 months, 3 months, and 1 year after transplant.

## Discussion

This is the largest study of Indigenous patients in the United States examining patient and allograft outcomes, dose-normalized tacrolimus concentrations, and hospitalizations and emergency room visits after KTx. We show that KTx outcomes for the Indigenous patients are similar to White recipients after accounting for baseline differences. Our study confirmed that the Indigenous patients who underwent KTx at our center had similar morbidity as the Indigenous patients with ESKD at large. Despite younger age, mean difference of 8 years between the groups, 44 Indigenous and 51 White patients had history of CV events, coronary artery disease with myocardial infarction followed by heart failure followed by stroke and peripheral vascular disease in descending order of frequency. After transplant, both groups experienced similar incidence of CV events. Several traditional and non-traditional risk factors have been identified as risk factors for CV events post-transplant, many were found to be more prevalent in our Indigenous KTx cohort such as: increased rates of diabetes, pre-transplant dialysis, obesity, and hypertension [20]. After accounting for these differences in baseline characteristics using propensity score matching, the CV event rates between the two groups were similar.

The U.S. Department of Health and Human Services Office of Minority Health has reported lower rates of cancer among the Indigenous patients with increased rates of subtypes of malignancy that differ by gender and age [21]. Racial differences in malignancy risk after KTx has been identified for Black and Hispanic recipients but has not been reported for the Indigenous patients post-transplant outside of skin [22]. The risk for malignancy in our cohort was significantly lower among the Indigenous patients and there were no cases of skin malignancy in that cohort which is consistent with known decreased prevalence of skin cancer in Indigenous KTx recipients compared to Whites after transplant [23]. The increase in malignancy risk after KTx has been partly attributed to baseline risk and in our study the White cohort had significantly higher rates of malignancy prior to KTx and after KTx consistent with current literature [22].

We showed that the Indigenous patients had a trend towards higher rates of infections, most commonly urinary tract infections. This could be explained by higher prevalence of dialysis and longer duration of dialysis for Indigenous patients compared to White. Deceased donor transplant and delayed graft function have been previously associated with increased rates of post-KTx urinary tract infections [24–26] and these factors were more prevalent for the Indigenous patients. After accounting for these variables in propensity matching, there was no difference noted in infectious complications.

The increased incidence of delayed graft function and acute rejection during the first year in the Indigenous patients compared to Whites was explained by the increased rates of deceased donor transplantation among the Indigenous patients. In the study by Kasiske and Chakkera, delayed graft function and rates of acute rejection were similar as the rates of deceased donor transplantation were also similar between the two groups [12]. In that study, the incidence of delayed graft function was remarkably higher among White patients at 38.9% compared to 15.8% in our cohort and the rates of deceased donor KTx were similar between the two groups (80.0% Indigenous versus 79.5% White) in contrast to our study where there was an almost 2 fold higher rate of deceased donor KTx among the Indigenous patients (82.4% versus 44.8% in Whites).

Zip code analyzes showed that the Indigenous patients resided further away from the transplant center but this data should be interpreted with caution as zip code data at the time of transplant was missing for 61.8% of the Indigenous patients and for 23.6% of Whites. In addition, the median follow-up time for the Indigenous patients was 2 years compared to 4 years for Whites. This may have affected our ability to capture outcomes of interest for the Indigenous patients beyond 2 years after transplant. It should be noted however that the range in years for follow-up was similar between the two groups. This also will affect interpretation of hospitalization rates as our study only captured hospitalizations and emergency room visits that occurred within our hospital and did not include health care utilization elsewhere. Therefore our finding of increased hospitalizations and emergency room visits after KTx for Whites compared to the Indigenous patients should be interpreted with caution.

Despite similar tacrolimus doses and trough levels, the Indigenous patients had higher dose-normalized tacrolimus trough concentrations compared to Whites particularly at 1 year post-transplant. This is consistent with recent report by Mohamed et al that compared dose normalized levels for patients from Indigenous, Asian, European, and African ancestry and showed that the Indigenous patients had the highest dose-normalized levels despite similar daily dose and trough levels [27] Patients who are carriers for the CYP3A5*3 variant are known to have higher dose-normalized trough concentrations and more Indigenous patients are known to be carriers for CYP3A5*3 variant compared to Whites which likely explains our findings of higher dose-normalized tacrolimus concentrations [27,28]. Our results therefore show that despite higher dose-normalized tacrolimus concentrations for the Indigenous patients, there was no significant difference in infectious complications or malignancy outcomes after transplant compared to Whites after adjustment for baseline differences.

The Indigenous American patients with ESKD have significantly lower rates of transplantation and these rates have been partially attributed to known risk factors: clinical, socioeconomic, and psychosocial [9–11]. Qualitative assessment has revealed other factors impacting perspective and experiences regarding KTx in general for the Indigenous patients [29]. We previously showed that lack of education was a primary barrier to transplantation [30]. Others have shown that fear of adverse outcomes after transplantation such as graft failure and return to dialysis is a common theme [31–33]. The results of this study provide objective evidence to support successful graft outcomes for the Indigenous patients. This may improve misconceptions about complications after kidney transplant and may increase engagement and interest in pursuing KTx. Future qualitative studies to assess for this are needed.

Several limitations warrant discussion. The findings of this study are representative of a single transplant center and the results may not be generalizable to other centers. However, our center serves a large population of Indigenous patients in Network 15 and similar studies are needed in other transplant centers that serve this population. The number of patients studied is small limiting further analysis, yet this remains the largest study to date with detailed clinical outcomes of Indigenous patients after KTx. Racial descriptions were established from patient reported data in the medical record which can be inaccurate and may create potential bias due to non-representative sampling; however this would affect both groups studied equally. Lastly, our study did not take into account outcome events that could have taken place outside of our Transplant Center but this would have affected both groups equally.

We have shown in this study that the Indigenous patients who undergo KTx at our transplant center have similar patient survival and graft outcomes compared to Whites after accounting for baseline differences. They are less likely to develop malignancy post-transplant and the risk for infectious complications is not different compared to Whites after accounting for known risk factors. Our study provides objective data to support similar outcomes after KTx for the Indigenous patients compared to Whites to fuel acceptance and engagement in pursuing KTx as the preferred treatment option for ESKD in the Indigenous American community.

## Supporting information

S1 File Deidentified data spreadsheet. (XLSX)
